# One-Step Treatment for Upgrading Bleached Bamboo Pulp to Dissolving Pulp High Solvency in Green Alkali/Urea Aqueous Solution

**DOI:** 10.3390/polym15061475

**Published:** 2023-03-16

**Authors:** Jiao-Ping Shang, Pin Liang, Yun Peng, Ding-Feng Xu, Yi-Bao Li

**Affiliations:** Engineering Research Center of Jiangxi Province for Bamboo-Based Advanced Materials and Biomass Conversion, College of Chemistry and Chemical Engineering, Gannan Normal University, Golden Campus, Ganzhou 341000, China

**Keywords:** bamboo pulp, molecular weight, one-step method, high solvency

## Abstract

Bleached bamboo pulp, as a kind of natural cellulose, has received significant attention in the field of biomass materials due to its advantages of environmental protection and the abundance of raw materials. Low-temperature alkali/urea aqueous system is a green dissolution technology for cellulose, which has promising application prospects in the field of regenerated cellulose materials. However, bleached bamboo pulp, with high viscosity average molecular weight (*M*η) and high crystallinity, is difficult to dissolve in an alkaline urea solvent system, restraining its practical application in the textile field. Herein, based on commercial bleached bamboo pulp with high *M*η, a series of dissolvable bamboo pulps with suitable *M*η was prepared using a method of adjusting the ratio of sodium hydroxide and hydrogen peroxide in the pulping process. Due to the hydroxyl radicals being able to react with hydroxyls of cellulose, molecular chains are cut down. Moreover, several regenerated cellulose hydrogels and films were fabricated in an ethanol coagulation bath or a citric acid coagulation bath, and the relationship between the properties of the regenerated materials and the *M*η of the bamboo cellulose was systematically studied. The results showed that hydrogel/film had good mechanical properties, as the *M*η is 8.3 × 10^4^ and the tensile strength of a regenerated film and the film have values up to 101 MPa and 3.19 MPa, respectively. In this contribution, a simple method of a one-step oxidation of hydroxyl radicals to prepare bamboo cellulose with diversified *M*η is presented, providing an avenue for a preparation of dissolving pulp with different *M*η in an alkali/urea dissolution system and expanding the practical applications of bamboo pulp in biomass-based materials, textiles, and biomedical materials.

## 1. Introduction

Natural cellulose from cotton, wood, and bamboo is the most abundant renewable resource on Earth [1,2,3]. Natural cellulose is considered to be the ideal substitute for current petroleum-based polymer materials [4,5]. However, cellulose is difficult to dissolve in common solvents, resulting from its unique microcrystal structures and strong hydrogen-bonding networks of an intra- and intermolecular nature [6,7,8]. Currently, numerous dissolution systems of cellulose have been reported, such as NaOH/CS_2_ [9], N-methylmorpholine-N-oxide (NMMO) [10], ionic liquids (ILs) [11,12], alkali/urea [13,14], and lithium chloride/dimethylacetamide (LiCl/DMA_C_) [15,16]. Even though NaOH/CS_2_ is widely used to produce viscose, the process is time-consuming, has high energy consumption, and produces toxic by-products [17]. For solvents with a strong dissolving ability, including NMMO, LiCl/DMA_C,_ and ionic liquids, industrial applications are still hindered by the high cost and the difficulties in solvent recycling [18]. Compared with the previously mentioned dissolution system, the alkali/urea aqueous system is an environmentally friendly technology for cellulose dissolution with a fast physical dissolution process, including alkali/urea, alkali/thiourea, and alkali/urea/thiourea [19,20]. Moreover, the alkali/urea aqueous system has the advantages of convenient operation, environmental protection, low energy consumption, and a high performance price ratio. Moreover, the order of dissolution rate of cellulose in alkali/urea aqueous system is LiOH/urea > NaOH/urea > KOH/urea. Based on this, various regenerated cellulose materials (including cellulose films, fibers, and foams/sponges) have been prepared and widely applied to many fields, such as textiles [21,22], packaging [23], water treatment [24,25], electromagnetic shielding [26,27], biosensors [22], etc. However, the viscosity average molecular weight (*M*η) of cellulose has a great influence on cellulose dissolution [28], forming, and the mechanical properties of the regenerated cellulose materials. In an alkali/urea aqueous solution, high-*M*η cellulose has poor solubility, leading to the weak mechanical properties of the regenerated cellulose materials [19]. Despite the fact that low-*M*η cellulose possesses an excellent solubility, the prepared cellulose materials exhibit a weak tensile strength [29], low crystallinity, and orientation degree, resulting from insufficient chain entanglement and the low draft ratio of the short-chain cellulose during regeneration [30]. Therefore, controlling the *M*η of cellulose is a key prerequisite to its dissolution in the large-scale fabrication of regenerated cellulose materials [31].

*M*η has a significant impact on the solubility of cellulose and the mechanical properties of regenerated cellulose materials [32]. In order to improve the solubility of cellulose in alkali system, various methods, such as ball milling [33,34], steam explosion pretreatment [35] and enzymatic treatment [36], can achieve higher solubility by changing the degree of polymerization (DP) of cellulose. Compared with the above methods [37], the one-step treatment method with a strong oxidizing solvent can reduce the *M*η of cellulose reported in this manuscript; controllable depolymerization of the molecular weight of cellulose can be achieved. In 1993, cellulose products were prepared from bleached wood pulp by dissolving the pulp using a peroxide treatment [38,39]. Subsequently, hydrogen peroxide and related peroxy compounds used as bleaching agents are the most widely used in industry [40,41]. Peroxides can degrade and decolorize cellulose [42]. It has been confirmed that perhydroxyl anions are the primary bleaching moiety in alkaline hydrogen peroxide aqueous solutions [43]. The mechanism of the alkaline peroxide bleaching of cellulose is well elucidated [44]. Most of the literature indicated that the hydroperoxide anion (HO_2_^−^) is a strong nucleophile and reacts with the chromophores of the enone type in alkaline solutions. Furthermore, H_2_O_2_ can decompose and generate exceedingly reactive hydroxyl radicals in various conditions, including UV light, Fenton and photo Fenton, ozone, ultrasound, and hot alkaline conditions, that can rapidly oxidize various inorganic and organic compounds [37,45].

A one-step treatment for upgrading bleached bamboo pulp by adjusting the ratio of sodium hydroxide and hydrogen peroxide is presented in this study. A series of bamboo cellulose dissolving pulps is obtained. Bamboo cellulose, with controllable *M*η and high solvency, can be conducive to the development of the textile industry and improve economic benefits. Moreover, the mechanism of controlling cellulose *M*η in an alkaline-peroxide solvent is further studied. Various regenerated cellulose hydrogels/films were prepared in a coagulation bath of ethanol or citric acid. In addition, the relationship between the properties of the regenerated materials and the *M*η of bamboo cellulose is systematically investigated. The objective of this study was to establish the relevance between *M*η and the solubility of cellulose. The mechanical properties of the regenerated cellulose materials have also been studied. This understanding will enrich the basic theory of cellulose depolymerization and dissolution and expand the practical applications of cellulosic materials in the field of textile, energy, environment, and biomedical materials.

## 2. Materials and Methods

### 2.1. Materials

The bleach bamboo pulp (α-cellulose content 88%, 1.4 × 10^5^ (*M*η)) was provided by Ganzhou Hwagain Paper Co., Ltd. (Ganzhou, China) in China. Lithium chloride (LiCl, 99%, AR), N,N-dimethylacetamide (HPLC, 99.8%), methane sulfonic acid (CH_3_SOOH, 90%), solid blue BB salt (AR), dimethylsulfoxide (C_2_H_6_OS, DMSO, 99.0%, AR), sodium hydroxide (NaOH, 98.0%), urea (AR, 99.0%), ethylene glycol ((CH_2_OH)_2_, 99.7%, AR), glycerol (C_3_H_8_O_3_, 99.0%, AR), and lithium hydroxide monohydrate (LiOH·H_2_O, 99.0%) were offered by Shanghai Titan Scientific Co., Ltd. (Shanghai, China) 30% hydrogen peroxide (H_2_O_2_, AR), ethanol (C_2_H_5_OH, 99.7%, AR), trisodium citrate (C_6_H_5_Na_3_O_7_, AR), citric acid monohydrate (C_6_H_8_O_7_, 99.5%, AR), disodium hydrogen phosphate (Na_2_HPO_4_, 99%), and hydrochloric acid (HCl, 30–38.0%, AR) were provided by Xilong Science Co., Ltd. (Shantou, China) Ethylenediamine copper (AR) was obtained by the China National Pulp and Paper Research Institute. All chemical reagents were used without further purification.

### 2.2. Preparation of Bamboo Cellulose Dissolving Pulp with Controllable Mη

An amount of 20 g of bleach bamboo pulp (BP) was soaked in 400 g of NaOH/H_2_O_2_ aqueous solutions with different solvent ratios in the autoclave (HT-1000KJ, Shanghai, China) at 100 °C and 0.08 MPa for 60 min. Then, a series of cellulose dissolving pulps with different *M*η was obtained after the treated BP was washed with deionized water and vacuum dried at 105 °C for 4 h. The dosage of NaOH varied from 0 to 6 wt%, and the dosage of H_2_O_2_ ranged from 0 to 1 wt%. The *M*η was coded as C14, C10, C8.3, C6.7, C5.1, and C4.0, which correspond to cellulose with *M*η of 14 × 10^4^, 10 × 10^4^, 8.3 × 10^4^, 6.7 × 10^4^, and 4.0 × 10^4^, respectively.

### 2.3. Preparation of Different Mη Bamboo Cellulose Solution

The 95 g LiOH·H_2_O/urea/deionized water (8:15:77 by weight) solution was pre-cooled to −12.6 °C. An amount of 5 g dried cellulose sample was fleetly added into solvent system and vigorously stirred for 2–5 min, the dissolved bamboo cellulose solution was obtained. The clear solutions with different *M*η were obtained after high-speed centrifugation (8000 rpm) of dissolved bamboo cellulose solution at 5 °C for 10 min.

### 2.4. Preparation of Bamboo Cellulose Hydrogels and Films with Different Mη

The 5 wt% bamboo cellulose solution was spread on a flat glass surface to form a solution film of cellulose with a thickness of 0.5 mm. Then, the liquid film of bamboo cellulose solution was immersed into a coagulation bath (ethanol or citric acid) for 30 min. The cellulose hydrogels with different *M*η were obtained after gelation of bamboo cellulose aqueous solution. Moreover, the dry films of bamboo cellulose were prepared by washing with deionized water and drying the cellulose hydrogel at room temperature.

### 2.5. Characterization

The viscosity average molecular weight (*M*η) was measured using an automatic viscometer (IV8100X, Hangzhou, China). An amount of 0.14 g of the prepared bamboo cellulose was added into 25 mL of deionized water and stirred for 10 min, forming the cellulose dispersion. Then, a completely dissolved cellulose solution was prepared after slowly adding 25 mL of copper ethylenediamine into the cellulose dispersion and stirring for 10 min. The intrinsic viscosity ([η]) of the bamboo cellulose was determined via a viscometer at 25 °C according to the following equation [46].

(1) $${\mathrm{DP}}^{0.905}=0.75[\eta ]$$

The molecular weight distribution (*D_Mw/Mn_*) of the bamboo cellulose was measured by liquid chromatography (e2695, Milford, MA, USA)/(2414RI, Waters, Milford, MA, USA). A mixed solution containing 0.025 g of bamboo cellulose and 3 mL of DMAc solvent was prepared and activated at 150 °C for 60 min. Then, LiCl (8 wt%) was added into the mixed solution at 100 °C for 60 min and maintained at 50 °C until the bamboo cellulose completely dissolved. Furthermore, the homogeneous solution was diluted to 0.5 wt% LiCl/DMAc with DMAc. The solution was filtered with a 0.22 µm Millipore filter membrane, and the test conditions were as follows: a flow rate of 0.6 mL/min, a column temperature of 80 °C, and a detector of 50 °C [47].

**Figure 1:**
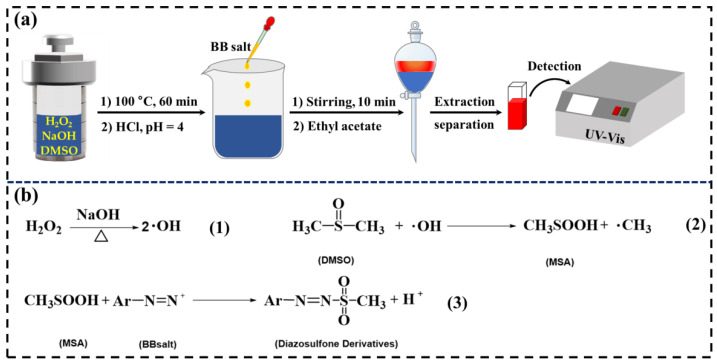
(**a**) The flowchart of the formation and detection of the ·OH. (**b**) Chemical equations in the process of quantitative detection of the ·OH.

A quantitative study of hydroxyl radical (·OH) derived from alkaline solution by UV-Vis absorption spectroscopy was performed. As shown in Figure 1a, the mixed aqueous solution consisting of NaOH (4, 12 and 24 g), H_2_O_2_ (0, 0.4, 1, and 2 g), and 23.4 g of DMSO was reacted at 100 °C for 60 min. Then, 1 mL of the reacted solution was diluted to 500 mL with pH = 4. Moreover, 1 mL of BB salt was added to 2 mL of the diluted solution and reacted for 10 min at room temperature. The diazo sulfone derivatives were extracted, separated, and detected by UVs spectrum (UV-1800, Shimadzu, Japan) at 415 nm in a range of 350–800 nm and a standard curve of CH_3_SOOH solution [48,49]. The chemical reactions in the process of the ·OH quantitative detection are shown in Figure 1b. The production of hydroxyl radicals in a high-temperature alkali/H_2_O_2_ system is shown in chemical Equation (1). DMSO, as a radical scavenger, captured the ·OH to produce methane sulfinic acid (MSA), as shown in chemical Equation (2). Simultaneously, the diazo sulfone derivatives were formed through the reaction of MSA and Blue BB salt (chemical Equation (3)).

Thermal gravimetric analyses (TGA) and derivative thermogravimetry (DTG) were carried out with a Q50 thermal analyzer (NETZSCH Corp, Selb, Germany) in air with a heating range from 25 to 600 °C by 10 °C/min. Solid-state ^13^C (Avance Neo, 400WB, Bruker, Billerica, MA, USA) cross-polarization magic-angle spinning (^13^C CP/MAS NMR) spectra were recorded with a 4 mm double-resonance MAS probe. A sample spinning rate of 10.0 kHz, a contact time of 2 ms, and a pulse delay of 3 s were applied. The chemical compositions of the before and after prepared bamboo pulp were characterized with an Avatar Fourier Transform Infrared Spectrometer (FT-IR, Nicolet Company, Madison, WI, USA).

The crystal peaks of the bamboo cellulose and the films with different *M*η were determined using an X-ray polycrystalline diffractometer (D8, ADVANCE, Karlsruhe, Baden-Württemberg, Germany) with the conditions of the voltage, current, scanning range, and scanning rate being 40 kV, 40 mA, 2*θ* = 5–40°, and 5°/min, respectively. The crystallinity ($\chi_{C}$) was calculated according to the ratio of the crystallization peak area to the integral area of the X-diffraction intensity curve of the cellulose samples and analyzed by the following Equation (2):

(2) $$\chi_{C}=\frac{S_{a}}{S_{a}{+S}_{c}}\times 100\%$$

where S_a_ and S_c_ are the crystalline areas and amorphous phases, respectively. The curve areas of the peaks were determined by integration, and they were recorded as the percentage of the crystalline peaks over the total area.

The topography dimensions of the bamboo celluloses with different *M*η were observed by environmental scanning electron microscopy (SEM, QUANTA 450, Gravenhage, South Holland, The Netherlands) under a voltage of 20 kV. The dissolution of the prepared bamboo cellulose samples with various *M*η was systematically investigated using a polarized light microscope (Axiolab5, Zeiss, Oberkohen, Battenrunsberg, Germany) at room temperature. The viscosity of the cellulose solution was measured through a rotary viscometer (HAAKE, Viscotester3, Karlsruhe, Baden-Württemberg, Germany). The solubility of the bamboo cellulose was investigated via a weighing method (Equation (3)), and the specific operations were as follows: (i) forming a cellulose solution layer on flat glass surface substance, (ii) regeneration of cellulose solution in the 5 wt% H_2_SO_4_ coagulation bath, and (iii) washing with deionized water to neutral and drying in the infrared oven for 10 min (WS70-1, Shanghai, China).

(3) $$A=\frac{M_{2}}{M_{1}}\times 100\%$$

where M_1_ is the mass of the added BP cellulose solution, M_2_ is the dried sample, and A is the solubility.

The particle size distributions of the diluted bamboo cellulose solutions were investigated by dynamic light scattering (DLS, 90 PALS, Brooke, Brooklyn, New York, USA). The bamboo cellulose solutions (*c* = 0.3 g/L) were filtrated through 0.45 μm Millipore filters. The hydrodynamic radius (*R*_h_) was calculated according to the following Stokes–Einstein, Equation (4) [50].

(4) $$R_{h}=\frac{\mathrm{KT}}{{6\pi \eta D}_{T}}$$

where T is the temperature, K is the Boltzmann constant, η is the viscosity of solvent, and D_T_ is the translational diffusion coefficient.

The rheological behaviors of the different *M*η solutions were characterized using a rheometer (HAAKE, RheoStress 600, Boston, MA, USA) with a gap of 500 μm from 20 °C to 80 °C. The cross-section morphologies of the cellulose hydrogels with different *M*η were observed by SEM (Zeiss, Sigma 500, Oberkohen, Battenrunsberg, Germany) at an accelerating voltage of 10 kV. The strain–stress curves of the hydrogels and films were measured by means of a universal material testing machine (INSTRON5965, Boston, MA, USA).

## 3. Results and Discussion

### 3.1. Effects of Alkaline Peroxide Treatment for Bamboo Cellulose Mη

**Figure 2:**
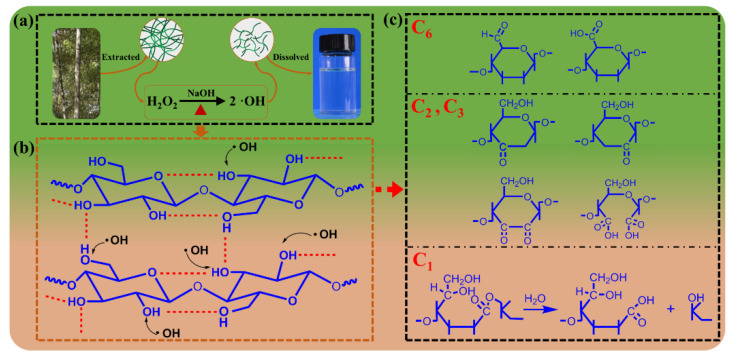
(**a**) Bamboo pulp with high *M*η converted into low bamboo pulp with high solubility through one-step processing (**a**). (**b**) The reaction of ·OH with hydroxyl groups in cellulose chains and various cellulose oxidation products (**c**).

A schematic diagram of the bleached bamboo pulp upgrades to a high-solvency bamboo pulp in the alkali system by regulating *M*η with OH originating from NaOH/H_2_O_2_ is shown in Figure 2a. As shown in Figure 2b, hydroxyl radicals are able to react with hydroxyl groups at C6, C3, C2, and C1 of the cellulose chains in the alkaline solution system [51,52,53] and form various cellulose oxidation products (Figure 2c). [37,54]. Consequently, the bamboo cellulose with lower *M*η after de-polymerization has higher solubility in the alkali/urine system.

**Figure 3:**
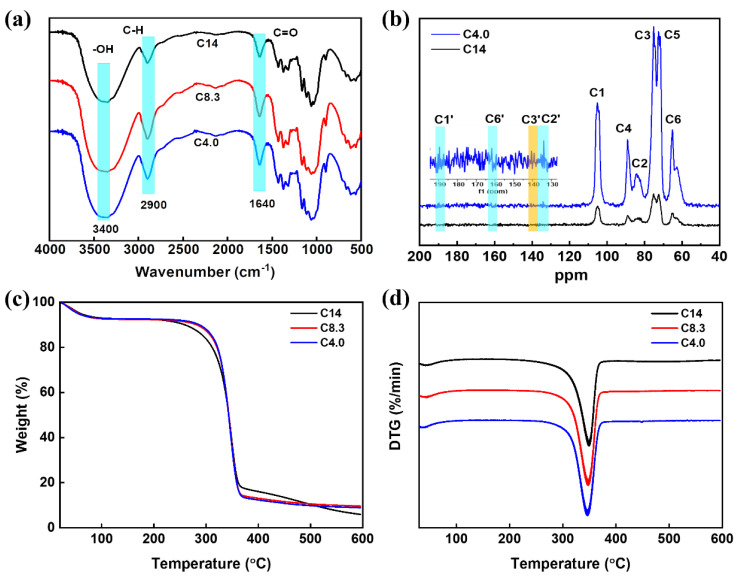
(**a**) FT-IR spectra of untreated (C14) and treated (C8.3 and C4.0) bamboo pulps. (**b**) Solid-state ^13^C CP/MAS NMR spectrum of untreated (C14) and treated (C4.0) bamboo pulps. The curves of TGA (**c**) and DTG (**d**) of untreated (C14) and treated (C8.3 and C4.0) bamboo pulps under a nitrogen atmosphere.

The Fourier-transform infrared spectroscopy (FT-IR) of the bamboo pulps with different *M*η (C14, C8.3, and C4.0) is shown in Figure 3a. The characteristic peaks at 3400 cm^−1^, 2900 cm^−1^, and 1640 cm^−1^ correspond to the O-H stretching vibration, C-H stretching vibration, and C=O stretching vibration, respectively. Evidently, the peak of C=O was significantly enhanced after the NaOH/H_2_O_2_ treatment, resulting from the formation of C=O groups that originated from the hydroxyls at C2, C3, and C6. Subsequently, the bamboo pulps of C4.0 and C14 were investigated by using the solid-state ^13^C CP/MAS NMR spectrum. As shown in Figure 3b, the signals that appeared at δ105.3, δ89.1, δ84.6, δ75.1, δ72.6, and δ65.0 in the spectrum correspond to the C1, C4, C2, C3, C5, and C6 of the glucose, respectively. Comparing the spectra of the untreated and treated samples, three weak C=O signal peaks appeared at δ161.9, δ140.8, and δ134.1 in the treated samples, which are responding to the C=O groups at C6′, C3′, and C2′. This result indicated that the hydroxyl groups at corresponding positions on cellulose chains were partially oxidized. The thermostability of the bamboo pulps with different *M*η (C14, C8.3, and C4.0) was evaluated by TGA and DTG. In Figure 3c,d, the maximum decomposition temperatures of the untreated and treated samples were 351.86 °C, 346.67 °C, and 341.24 °C, respectively. Due to the formation, decomposition, and evaporation of the C=O groups originating from the hydroxyls at C2, C3, and C6, an advanced loss in weight was caused.

**Figure 4:**
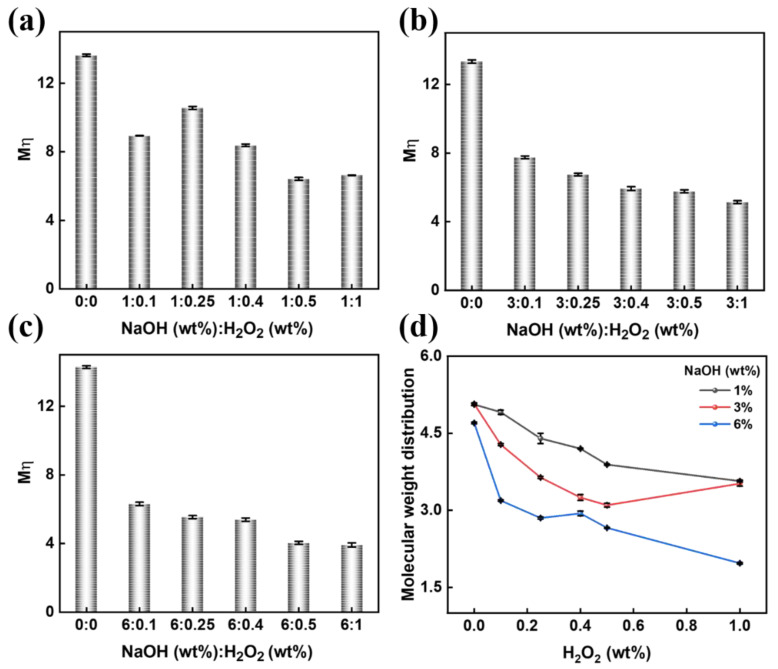
The effect of H_2_O_2_ concentration (from 0 to 1 wt%) on *M*η in the concentration of NaOH, including 1 wt% (**a**), 3 wt% (**b**), and 6 wt% (**c**). (**d**) *D_Mw/Mn_* of bamboo cellulose in different NaOH/H_2_O_2_.

The processability of bamboo pulp is typically determined by their solubility. *M*η is one of the most important parameters for the efficient dissolution of bamboo pulps. Regulating the *M*η of bamboo cellulose with different dosages (mass fraction) of NaOH and H_2_O_2_ is displayed in Figure 4a–c. Compared with only a NaOH solution, *M*η is easier to regulate by a mixed solution of NaOH and H_2_O_2_ treatment. The ·OH was able to oxidize hydroxyl groups of cellulose in the position of the C1, C2, C3, and C6, resulting in the de-polymerization of the cellulose and forming lower *M*η cellulose. Generally, as a fixed dosage of NaOH (1wt%, 3wt%, and 6 wt%), the amount of ·OH increased with the increased concentration of H_2_O_2_ (from 0~1 wt%). Similarly, the number of hydroxyl radicals produced at different NaOH concentrations was in disaffinity. Significantly, the amount of reactive ·OH was less than that in real production, because ·OH is easily quenched at different alkali concentrations. The results showed that the *D_Mw/Mn_* of the bamboo cellulose was determined by the synergistic effect of the mixed solution of NaOH and H_2_O_2_, as seen in Figure 4d. For instance, the *D_Mw/Mn_* could be changed in the range of 4.20 to 2.99, as in a mixed solution consisting of 0.4 wt% H_2_O_2_ and NaOH with different dosages. The alkali peeling reaction on the bamboo cellulose reacted by means of the outside-to-inside, causing the *D_Mw/Mn_* to decline slowly after the reaction speed was accelerated by using increasing alkali concentrations. Moreover, the results obtained by adjusting the dosages of NaOH and H_2_O_2_ showed that both the *M*η and *D_Mw/Mn_* of the prepared bamboo pulps declined (Table S1).

**Figure 5:**
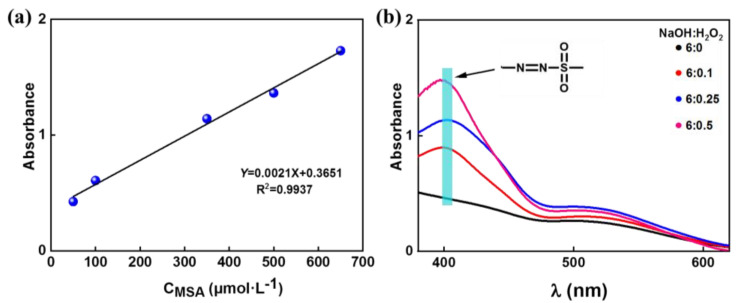
(**a**) The standard curve of *C*_(MSA)_ and (**b**) absorbance curve of diazo derivatives.

A standard curve of the relationship between the concentration of MSA (*C*_MSA_) and absorbance is presented in Figure 5a. The formula was *Abs* = 0.0021 × C_MSA_ + 0.3651, with R^2^ = 0.9937. Based on this, the total amount of ·OH was calculated, as shown in Figure 5b. The absorbance was up to 1.486 in the mixed solution of 6 wt% NaOH and 0.5 wt% H_2_O_2_, and the absorbance increased with the increasing H_2_O_2_ mass fraction. Moreover, this linear rule was also compounded in the mixed solution containing 1 wt% NaOH and 3 wt% NaOH systems (Figure S1). Due to the fact that the detected products had a short lifetime and that the reactive radical species were difficult to detect, the detected data are approximate values in the three cases. The relationship between the amount of ·OH and *M*η is shown in Figure S2: the more·OH produced, the lower the *M*η.

### 3.2. Solubility of Different Mη Bamboo Cellulose in LiOH/Urea/Aqueous Solution

The physicochemical parameters of the resultant bamboo cellulose with different *M*η are displayed in Table 1. The results showed that the parameters of the α-cellulose content and the whiteness increased, the hemicellulose content and the cellulose yield decreased, and the other indexes (ash, Fe^3+^, and dichloromethane extract) changed slightly. However, *χ_c_* exhibited a trend of first increasing and then decreasing, resulting from the fact that the degree of damage to the crystalline and amorphous regions of the cellulose was different during the alkaline oxidation treatment. Thus, cellulose dissolution required unwrapping and devitrification, causing the crystallinity to affect the cellulose dissolution in an alkali/urea aqueous solvent.

**Figure 6:**
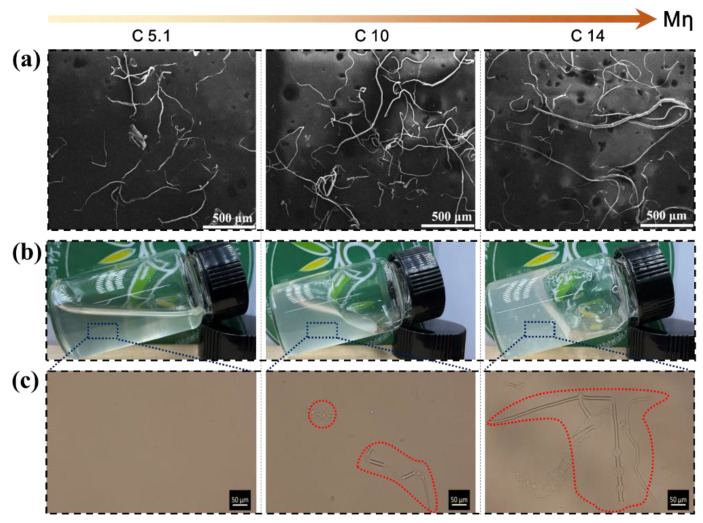
(**a**) The SEM images of bamboo cellulose with the *M*η of C5.1 and C10 and C14. (**b**) Optical pictures of bamboo cellulose solutions with the *M*η of C5.1 and C10 and C14. (**c**) Optical microscope images of bamboo dissolving solutions with the *M*η of C5.1 and C10 and C14.

As shown in Figure 6a and Figure S3a, the morphologies of the prepared bamboo cellulose with different *M*η were characterized by SEM. The micron-scale bundle chain fibers of bamboo cellulose were clearly observed, and the length of the bundle chain fibers decreased with decreasing *M*η. The original length of the bleached bamboo cellulose was about 3.09 mm (C14), and the length of the resultant bamboo cellulose was in the range of 1.32 mm (C10) to 0.28 mm (C4.0), as seen in Table S2. The optical photographs of the bamboo cellulose solutions with different *M*η in the LiOH/urea aqueous system are presented in Figure 6b and Figure S3b. Obviously, in the LiOH/urea aqueous system, the cellulose dissolved solution with low *M*η (C5.1) has better fluidity than the high *M*η (C14) dissolved solution. In addition, the optical microscope images of the cellulose dissolved solution in Figure 6c and Figure S3c further confirmed that the low *M*η fibers were completely dissolved, forming a uniform transparent solution with good fluidity. In contrast, the vast majority of the longer fibers were undissolved, forming a high viscosity or low flow-ability cellulose solution; this is due to the entanglement between the hydroxyl groups of the longer bundle chain fibers.

**Figure 7:**
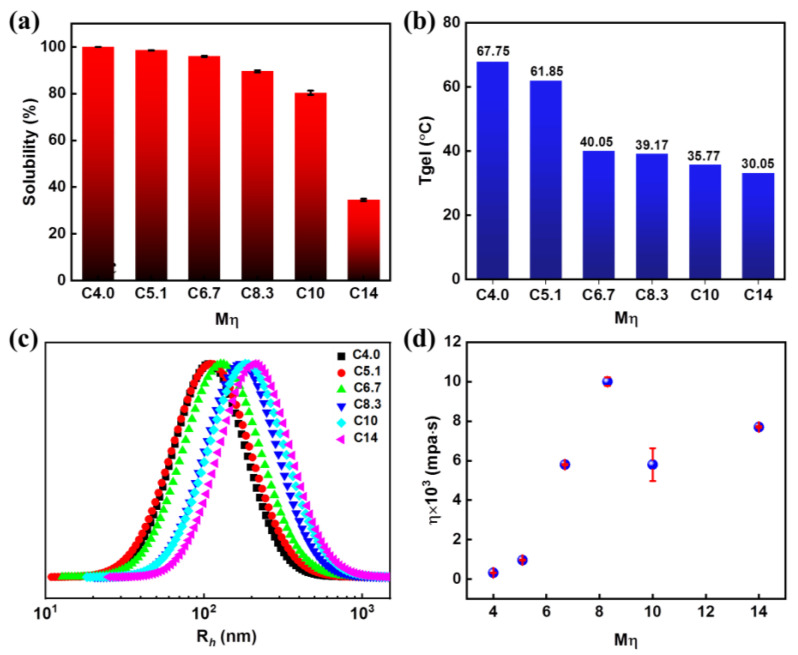
The solubility (**a**), gel temperature (**b**), DLS curves (**c**), and viscosity (**d**) of bamboo cellulose with different *M*η in LiOH/urea aqueous solutions.

The relationship between the solubility and the *M*η of the bamboo cellulose in the LiOH/urea aqueous system was investigated, as shown in Figure 7a and Table S3. The solubility of the bamboo cellulose decreased from 100% to 34.5% with increased *M*η from C4.0 to C14. Moreover, the stability of the dissolved solution of the bamboo cellulose with different *M*η was further studied by means of analyzing rheology, as shown in Figure 7b and Figure S4. The results showed that the gel point decreased from 67.75 °C to 33.05 °C with increasing *M*η (Figure 7b) because the thermal motion and the effective collision of the cellulose molecule chains were increased with increasing temperatures. In addition, the loss modulus (*G″)* and storage modulus (*G′*) increased with the increasing *M*η during the heating process from 20 °C to 80 °C (Figure S4). Subsequently, DLS was used to observe the size of agglomerates of the bamboo cellulose solution with different *M*η, as shown in Figure 7c and Table S3. The *R_h_* increased from 106.8 nm to 215.72 nm, with *M*η increasing from C4.0 to C14. The formation of large aggregates was due to increased cellulose molecular weight. Furthermore, the results of rotary viscometer of the prepared cellulose solution with different *M*η were shown in Figure 7d. The viscosity of the bamboo cellulose increased from 323.4 mPa·s to 12,449.8 mPa·s with increasing *M*η from C4.0 to C8.3. However, the viscosities at C10 and C14 were less than the highest value at C8.3. Due to bamboo celluloses (C10 and C14) having massive hydroxyl groups and chain entanglement, the solution viscosity is increased and difficulties in separation by centrifugation arise.

### 3.3. Structure and Mechanical Properties of Hydrogels and Dry Films

The structure-mechanical properties relationships of the regenerated cellulose materials with different *M*η were investigated. The cross-section SEM images of the regenerated cellulose materials, both in ethanol coagulation bath (Figure S5a–e) and in citric acid coagulation bath (Figure S5f–j), were generated. The *M*η ranged from C5.1, C6.7, C8.3, and C10 to C14, respectively. Due to the lowest viscosity and good flowability of the cellulose solution at C4.0, the regenerative cellulose material was uniform. It was difficult to characterize the actual performance of the materials, as shown in Figure S6a,b. The different *M*η of the bamboo cellulose caused different amounts of hydroxyl groups in the cellulose chain. Furthermore, several structures of the regenerated cellulose materials were formed through the parallel aggregation of intra- or intermolecular hydrogen bonds. The bamboo cellulose solutions with high dissolution rates in the alkali/urea system, such as C5.1, C6.7, and C8.3, formed regenerated materials with a dense porous/layer structure. On the contrary, the cellulose solution with low solubility (C10 and C14) formed porous-structured materials. Moreover, the regeneration rate of the cellulose solution in ethanol was slower than citric acid and formed cellulose with a denser structure.

**Figure 8:**
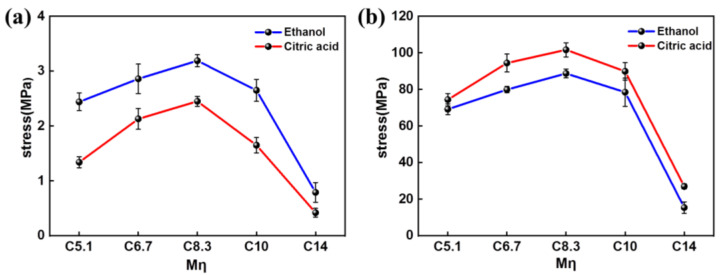
The tensile stress curves of hydrogels (**a**) and films (**b**) with *M*η of C5.1, C6.7, C8.3, C10, and C14.

The mechanical properties of the regenerated cellulose materials (hydrogels and dry films) with different *M*η were investigated. Figure 8a summarizes the tensile stress of the cellulose hydrogels in different coagulation baths, including ethanol and citric acid. The result indicated that the stress of the cellulose hydrogel in ethanol was higher than in citric acid. This phenomenon corresponded to the structure of cellulose materials (in Figure S5), in which the greater stress comes from the denser structure. Moreover, the fracture of the stress–strain curve increased gradually from 2.44 to 3.19 MPa and 30.64% to 41.78% with increasing *M*η (C5.1 to C8.3) in the ethanol coagulation bath. The relationship between the *M*η and the stress–strain is exhibited in Figure S7a, and the mechanical properties of the regenerated cellulose hydrogels are summarized in Table S4. Furthermore, as shown in Figure 8b, the tensile stresses of the cellulose films in two coagulation baths (ethanol and citric acid) with the same *M*η (C8.3) are presented. The results showed that the stress of the regenerated cellulose film in citric acid was higher than in ethanol. This phenomenon is contrary to the mechanical properties of the cellulose hydrogels, resulting from the reorganization of the internal structure caused by the occurrence of moisture. In addition, the stress–strain of the regenerated cellulose films increased first and then decreased, similar to the trend seen with the cellulose hydrogels. The stress–strain of cellulose was maximal at values of 101.66 MPa and 5.69 %, with a *M*η vaue of C8.3 (Figure S7b and Table S5). Therefore, the corresponding cellulose solution has good fluidity and the regenerated cellulose materials have excellent mechanical properties.

The XRD data of the regenerated cellulose film are presented in Figure S8a,b. The tested samples produced three characteristic peaks at 12°, 20°, and 22.3° (2*θ*), corresponding to the (10), (1$\bar{1}$0), and (200) planes of the cellulose II crystalline form, respectively. The crystallinity ($\chi_{C}$) of the cellulose film regenerated in citric acid first increased (47.82% to 60.49%) and then decreased (60.49% to 42%), and the highest value of 60.49% corresponded to C8.3. Moreover, the $\chi_{C}$ of the cellulose films regenerated in ethanol also first increased and then decreased with increased cellulose *M*η. The highest value of $\chi_{C}$ is 49.69% (C10), as shown in Figure S8c. The results demonstrate that the bamboo cellulose dry films have a higher $\chi_{C}$ when regenerated in citric acid than in ethanol, corresponding to the mechanical properties of the cellulose film. Therefore, these results could confirm that crystallinity is also an important parameter for mechanical performance and that only cellulose with appropriate *M*η has a good crystallinity.

## 4. Conclusions

In conclusion, a one-step method of the treatment of NaOH/H_2_O_2_ to regulate the *M*η of commercial, bleached bamboo pulp with high *M*η, realizing the preparation of various bamboo pulps with a low *M*η (C4.0, C5.1, C6.7, C8.3, and C10) and efficient dissolving in a LiOH/urea aqueous solution, was developed. Moreover, the solution concentration of NaOH/H_2_O_2_ was systematically studied, indicating that the higher the concentration, the lower the molecular weight. In addition, the reason for the decreased *M*η of the bamboo cellulose was revealed, resulting from the effect of the reaction of ·OH with the hydroxyl groups on the bamboo cellulose. Interestingly, the mechanical properties of the regenerated bamboo cellulose materials increased with increased *M*η (*M*η < C10), as the *M*η of C8.3 had the best mechanical properties; the tensile stress of bamboo cellulose film was up to 101 MPa and the strength of the bamboo cellulose hydrogel was about 3.19 MPa. In this work, a simple method to prepare bamboo cellulose with diversified *M*η is presented, and the regenerated cellulose materials with appropriate *M*η possess excellent mechanical properties. It is believed that this study can provide significant guidance for creating a cellulose pulp with a suitable *M*η for an alkali/urea aqueous system. This study also expands the practical applications of bamboo pulp in biomass-based materials, textiles, and biomedical materials.

## Figures and Tables

**Table 1:** The crystallinity and α-cellulose of the prepared bamboo cellulose with different *M*η.

*M*η (w)	α-Cellulose (%)	Hemicellulose (%)	Ash (%)	Fe^3+^ (mg/kg)	Whiteness (%)	Dichloromethane Extract (%)	Yield (%)	*χ_c_* (%)
C4.0	/	3.2 ± 0.45	1.0 ± 0.01	58.8 ± 1.25	86.6 ± 1.83	4.8	85.0	60.0
C5.1	90.0 ± 0.01	7.0 ± 0.67	1.1 ± 0.02	58.8 ± 1.71	87.0 ± 2.31	4.7	87.0	48.5
C6.7	89.0 ± 0.21	7.1 ± 0.54	1.0 ± 0.04	59.4 ± 1.83	87.0 ± 1.48	5.8	90.0	47.3
C8.3	88.4 ± 0.11	9.7 ± 0.38	0.9 ± 0.01	62.4 ± 2.01	86.0 ± 1.38	5.6	92.0	49.1
C10	87.5 ± 0.17	9.7 ± 0.34	1.1 ± 0.03	61.2 ± 2.23	85.0 ± 1.54	5.9	94.0	53.2
C14	86.0 ± 0.01	14.2 ± 0.43	1.1 ± 0.04	60.3 ± 1.34	84.0 ± 1.67	6.0	/	57.2

## Data Availability

The data presented in this study are available on request from the corresponding author.

## Supplementary Materials

The following supporting information can be downloaded at: https://www.mdpi.com/article/10.3390/polym15061475/s1, Table S1: The *M*η and *D_Mw/Mn_* of bamboo pulps treated with NaOH and H_2_O_2_ solutions of different mass fractions; Figure S1: The absorbance of diazo salt changed with the increase of H_2_O_2_ under the conditions of 1 wt% NaOH (a) and 3 wt% NaOH (b); Figure S2: The relationship between the hydroxyl radicals and the *M*η of bamboo pulp in three alkali conditions: 1 wt% (a), 3 wt% (b) and 6 wt% (c); Figure S3: (a) The SEM images of bamboo cellulose with different *M*η. Optical pictures (b) and optical microscope photographs (c) of bamboo cellulose solutions with different *M*η; Table S2: The *M*η of bamboo pulps are corresponding to cellulose bundle chain size; Table S3: Solubility and DLS of bamboo cellulose solutions with different *M*η; Figure S4: Gel temperature of bamboo cellulose solutions with different *M*η; Figure S5: The cross-section SEM images of the regenerated cellulose materials in ethanol coagulation bath (a–e) and citric acid coagulation bath (f–j); Figure S6: The regenerated cellulose hydrogels of C4.0 (*M*η, 1.0 × 10^4^) in ethanol coagulation bath (a) and citric acid coagulation bath (b); Figure S7: The stress–strain curves of regenerated hydrogels (a) and dry films (b) of bamboo cellulose with different *M*η; Table S4: The stress–strain data of the regenerated cellulose hydrogels with different *M*η; Table S5: The stress–strain data of the regenerated bamboo cellulose films with different *M*η; Figure S8: The XRD patterns and crystallinity of regenerate films in two coagulation baths. (a) In ethanol coagulation bath. (b) In citric acid coagulation bath. (c) The crystallinity of regenerated films in two coagulation baths.
