# Supplementary material for: One-Step Treatment for Upgrading Bleached Bamboo Pulp to Dissolving Pulp High Solvency in Green Alkali/Urea Aqueous Solution
Source: Polymers (Basel). 2023 Mar 16;15(6):1475. doi: 10.3390/polym15061475 (PMC10056472; doi:10.3390/polym15061475)
Supplement: Supplementary file 1 [file polymers-15-01475-s001.zip › polymers-2254271-supplementary.pdf]

Supporting Information

One-step treatment for upgrading bleached bamboo pulp to dissolving pulp high solvency in green alkali/urea aqueous solution

Jiao-Ping Shang† Pin Liang†, Yun Peng*, Ding-Feng Xu and Yi-Bao Li*

Engineering Research Center of Jiangxi Province for Bamboo-based Advanced Materials and Biomass Conversion. School of Chemistry and Chemical Engineering, Gannan Normal University, Ganzhou, 341000, P. R. China.

Email: pengyun@buaa.edu.cn (Y.P.); liyb@gnnu.cn (Y.-B.L.)

Table S1. The *M*η and $D_{Mw/Mn}$ of bamboo pulps treated with NaOH and H_2O_2 solutions of different mass fractions.

NaOH(wt%):H_2O_2 (wt%)	*M*η×(10^4)	*M*n×(10^4)	*M*w×(10^4)	$D_{Mw/Mn}$
0:0	14.13±0.07	8.03±0.02	41.54±0.15	5.17±0.15
1:0	13.62±0.04	11.33±0.15	57.37±0.17	5.06±0.02
1:0.1	8.95±0.01	8.74±0.13	42.91±0.15	4.91±0.04
1:0.25	10.44±0.09	8.70±0.12	38.27±0.19	4.40±0.10
1:0.4	8.32±0.07	8.35±0.08	35.07±0.15	4.20±0.01
1:0.5	6.40±0.09	6.70±0.15	26.07±0.21	3.89±0.01
1:1	6.74±0.01	6.62±0.07	23.63±0.15	3.57±0.02
3:0	13.43±0.01	10.38±0.11	52.62±0.28	5.07±0.02
3:0.1	7.67±0.08	6.98±0.05	29.92±0.35	4.28±0.02
3:0.25	6.68±0.08	6.33±0.25	23.09±0.23	3.65±0.02
3:0.4	5.81±0.11	6.08±0.14	19.87±0.25	3.25±0.05
3:0.5	5.68±0.09	5.41±0.03	16.79±0.31	3.09±0.04
3:1	5.06±0.08	6.35±0.15	22.33±0.09	3.52±0.05
6:0	14.36±0.02	10.19±0.15	48.12±0.16	4.70±0.02
6:0.1	6.31±0.10	7.40±0.09	23.74±0.19	3.19±0.02
6:0.25	5.64±0.09	6.14±0.11	17.63±0.21	2.85±0.02
6:0.4	5.29±0.11	5.84±0.21	17.47±0.11	2.94±0.05
6:0.5	4.14±0.09	5.34±0.15	14.27±0.14	2.66±0.01
6:1	3.79±0.12	4.57±0.06	9.11±0.11	1.98±0.15

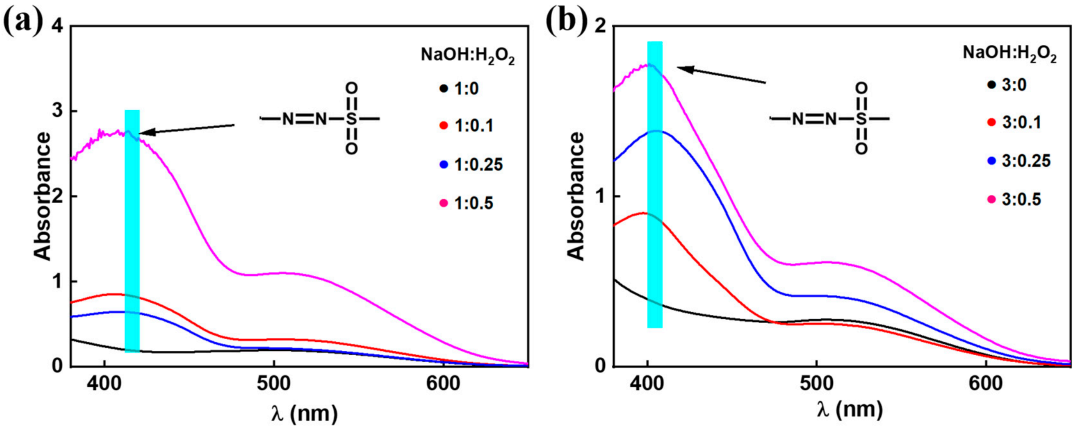

Figure S1. The absorbance of diazo salt changed with the increase of H_2O_2 under the conditions of 1 wt% NaOH (a) and 3 wt% NaOH (b).

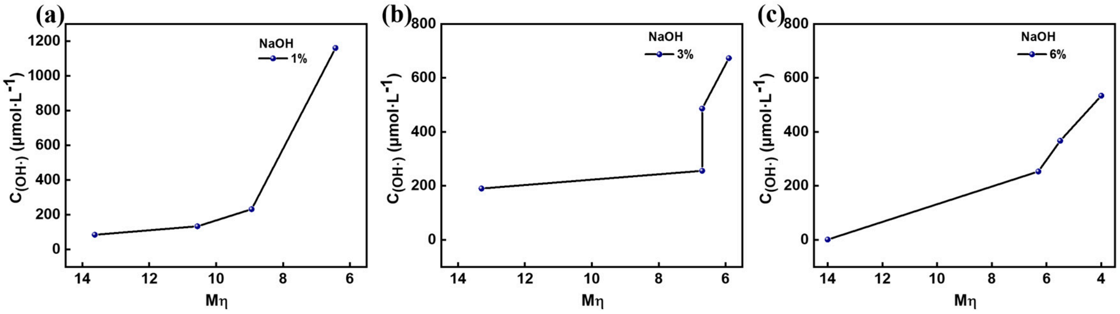

Figure S2. The relationship between the hydroxyl radicals and the *M*η of bamboo pulp in three alkali conditions: 1 wt% (a), 3 wt % (b) and 6wt% (c).

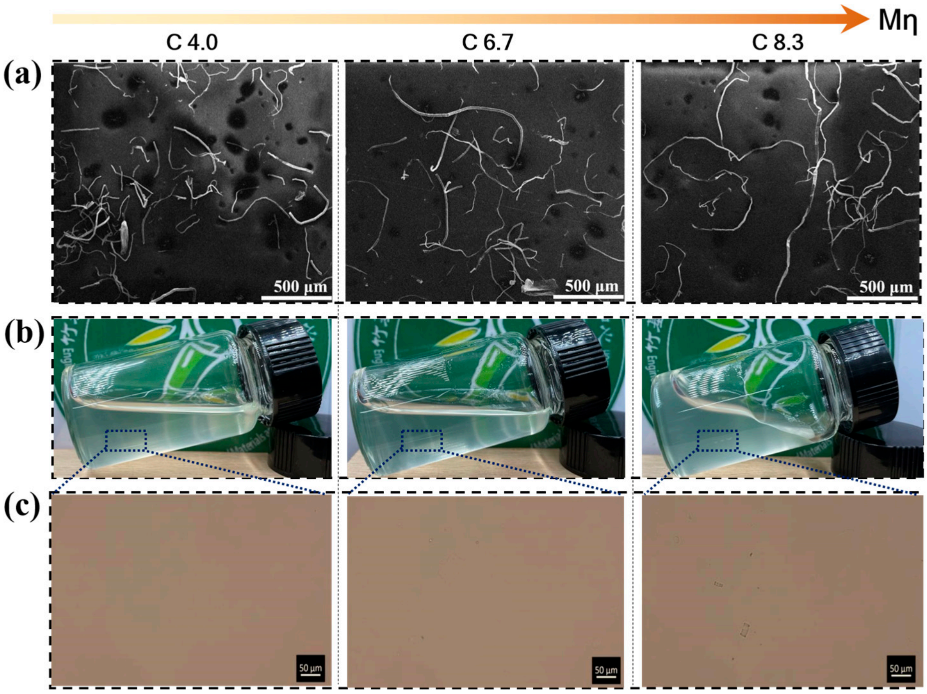

Figure S3. (a) The SEM images of bamboo cellulose with different Mη. Optical pictures (b) and optical microscope photographs (c) of bamboo cellulose solutions with different *M*η.

Table S2. The $M\eta$ of bamboo pulps are corresponding to cellulose bundle chain size.

$M\eta\times(10^4)$	C4.0	C5.1	C6.7	C8.3	C10	C14
**Fiber size（mm）**	0.28±0.01	0.57±0.02	0.67±0.08	0.98±0.04	1.32±0.06	3.09±0.03

Table S3. Solubility and DLS of bamboo cellulose solutions with different $M\eta$.

$M\eta\times(10^4)$	C4.0	C5.1	C6.7	C8.3	C10	C14
**Solubility(%)**	100±0.012	98.5±0.153	96.0±0.252	89.6±0.447	80.4±0.917	34.5±0.492
R_h**(mm)**	106.80	121.78	127.65	176.65	207.43	215.72

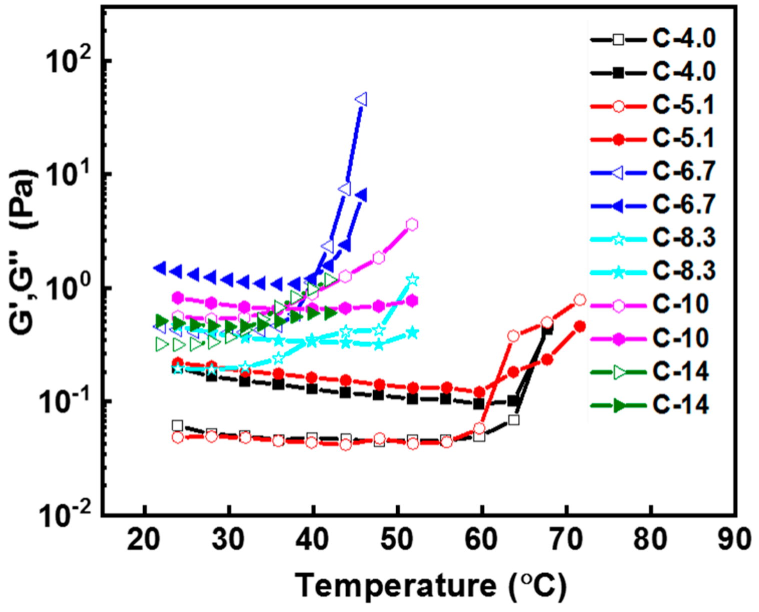

Figure S4. Gel temperature of bamboo cellulose solutions with different $M\eta$.

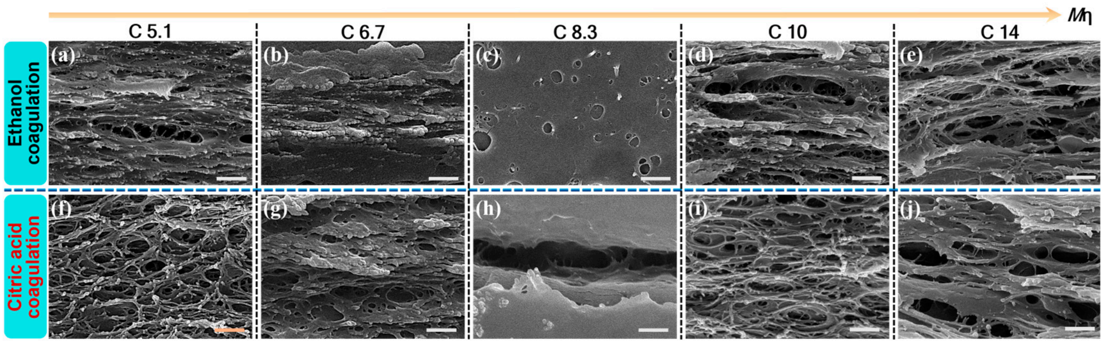

Figure S5. The cross-section SEM images of the regenerated cellulose materials in ethanol coagulation bath (a-e) and citric acid coagulation bath (f-j). Scale bar (a-j): 500 nm.

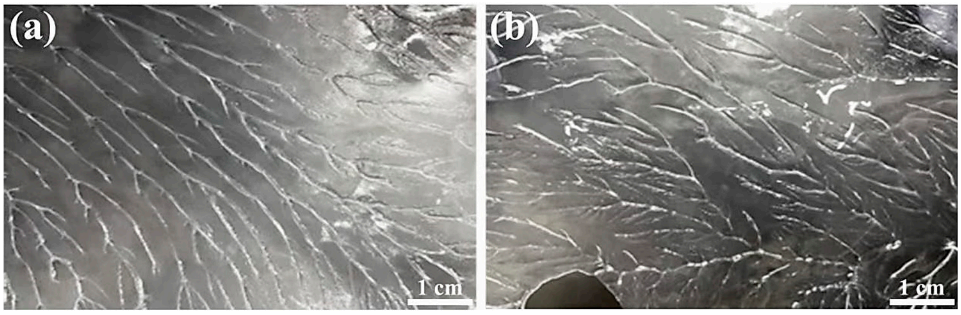

Figure S6. The regenerated cellulose hydrogels of C4.0 ($M\eta$, 1.0×10^4) in ethanol coagulation bath (a) and citric acid coagulation bath (b).

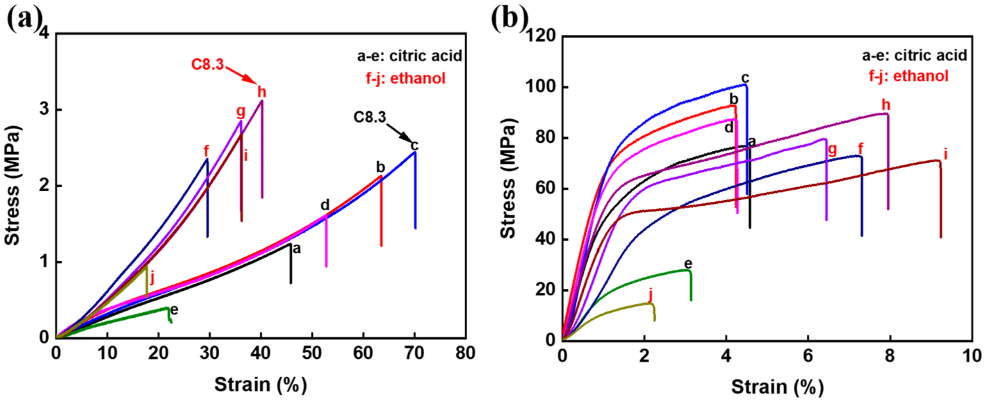

Figure S7. The stress-strain curves of regenerated hydrogels (a) and dry films (b) of bamboo cellulose with different $M\eta$.

Table S4. The stress-strain data of the regenerated cellulose hydrogels with different $M\eta$.

$M\eta\times(10^4)$	Coagulation bath	Modulus (MPa)	Stress (MPa)	Strain (%)
C5.1	Ethanol	10.70±0.46	2.44±0.16	30.64±0.82
C6.7		11.44±0.52	2.86±0.27	36.84±1.54
C8.3		11.33±0.09	3.19±0.11	41.78±1.36
C10		10.77±0.54	2.65±0.20	35.92±1.26
C14		5.68±0.86	0.79±0.18	16.36±2.53
C5.1	Citric acid	3.32±0.18	1.34±0.10	49.87±4.54
C6.7		4.99±0.34	2.13±0.19	63.92±3.54
C8.3		5.56±0.26	2.45±0.09	72.92±2.37
C10		4.37±0.37	1.65±0.14	53.71±2.93
C14		2.26±0.33	0.42±0.08	23.51±3.54

Table S5. The stress-strain data of the regenerated bamboo cellulose films with different *M*η.

*M*η×(10^4)	Coagulation bath	Modulus (MPa)	Stress (MPa)	Strain (%)
C5.1		4399.55±1039.49	69.19±2.89	5.73±1.92
C6.7		5206.93±481.11	79.91±1.70	7.13±0.32
C8.3	Ethanol	5743.4±668.97	88.74±2.44	7.72±0.72
C10		5172.04±421.69	71.39±7.70	5.73±1.15
C14		1190.1±436.40	15.39±3.12	3.02±0.78
C5.1		5885.59±157.35	74.40±3.39	4.97±0.99
C6.7		7478.11±307.85	94.43±4.94	5.26±1.00
C8.3	Citric acid	7527.28±675.00	101.66 ± 3.86	5.69±0.47
C10		7098.90±324.44	89.87±4.74	6.01±1.39
C14		1968.62±424.67	26.98±1.39	3.07±0.75

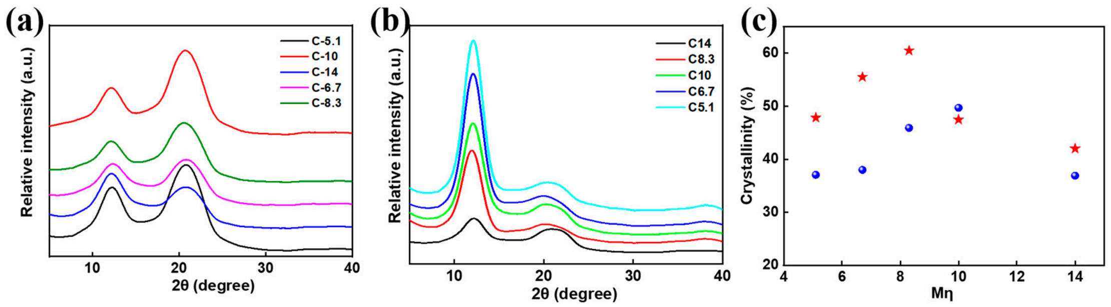

Figure S8. The XRD patterns and crystallinity of regenerate films in two coagulation baths. (a) In ethanol coagulation bath. (b) In citric acid coagulation bath. (c) The crystallinity of regenerate films in two coagulation baths.
